# Spatial distribution of HIV, HCV, and co-infections among drug users in the southwestern border areas of China (2004–2014): a cohort study of a national methadone maintenance treatment program

**DOI:** 10.1186/s12889-017-4769-7

**Published:** 2017-09-30

**Authors:** Mingli Li, Rongjian Li, Zhiyong Shen, Chunying Li, Nengxiu Liang, Zhenren Peng, Wenbo Huang, Chongwei He, Feng Zhong, Xianyan Tang, Guanghua Lan

**Affiliations:** 1 Institute of Vaccine Clinical Research, Guangxi Zhuang Autonomous Region Center for Disease Control and Prevention, Nanning, Guangxi 530028 China; 2 Institute of HIV/AIDS Prevention and Control, Guangxi Zhuang Autonomous Region Center for Disease Control and Prevention, 18 Jinzhou Road, Nanning, 530028 Guangxi People’s Republic of China; 3 School of Public Health, Guangxi Medical University, Nanning, Guangxi 530021 China

**Keywords:** Spatial distribution, HIV, HCV, Co-infections, Drug users, Methadone maintenance treatment

## Abstract

**Background:**

A methadone maintenance treatment (MMT) program to curb the dual epidemics of HIV/AIDS and drug use has been administered by China since 2004. Little is known regarding the geographic heterogeneity of HIV and hepatitis C virus (HCV) infections among MMT clients in the resource-constrained context of Chinese provinces, such as Guangxi. This study aimed to characterize the geographic distribution patterns and co-clustered epidemic factors of HIV, HCV and co-infections at the county level among drug users receiving MMT in Guangxi Zhuang Autonomous Region, located in the southwestern border area of China.

**Methods:**

Baseline data on drug users’ demographic, behavioral and biological characteristics in the MMT clinics of Guangxi Zhuang Autonomous Region during the period of March 2004 to December 2014 were obtained from national HIV databases. Residential addresses were entered into a geographical information system (GIS) program and analyzed for spatial clustering of HIV, HCV and co-infections among MMT clients at the county level using geographic autocorrelation analysis and geographic scan statistics.

**Results:**

A total of 31,015 MMT clients were analyzed, and the prevalence of HIV, HCV and co-infections were 13.05%, 72.51% and 11.96% respectively. Both the geographic autocorrelation analysis and geographic scan statistics showed that HIV, HCV and co-infections in Guangxi Zhuang Autonomous Region exhibited significant geographic clustering at the county level, and the Moran’s *I* values were 0.33, 0.41 and 0.30, respectively (*P* < 0.05). The most significant high-risk overlapping clusters for these infections were restricted to within a 10.95 km^2^ radius of each of the 13 locations where P county was the cluster center. These infections also co-clustered with certain characteristics, such as being unmarried, having a primary level of education or below, having used drugs for more than 10 years, and receptive sharing of syringes with others. The high-risk clusters for these characteristics were more likely to reside in the areas surrounding P county.

**Conclusions:**

HIV, HCV and co-infections among MMT clients in Guangxi Zhuang Autonomous Region all presented substantial geographic heterogeneity at the county level with a number of overlapping significant clusters. The areas surrounding P county were effective in enrolling high-risk clients in their MMT programs which, in turn, might enable people who inject drugs to inject less, share fewer syringes, and receive referrals for HIV or HCV treatment in a timely manner.

## Background

HIV and hepatitis C virus (HCV) infections are major global public health concerns, with overlapping routes of transmission, populations most affected and geographical areas. Data from 2014 suggested that more than 36.9 million people worldwide were living with HIV [1], 115 million people were estimated to be HCV antibody-positive [2], and approximately 2.3 million were estimated to have HIV/HCV co-infection, of whom 59% were people who inject drugs (PWID) [3]. Similar to other oversea countries, China has witnessed the fastest-growing HIV and HCV epidemics fueled by injecting drug users (IDUs) over the past three decades and is experiencing the highest burden of these infections in PWID at present [4–6]. Methadone maintenance treatment (MMT) programs were first initiated in China as a small pilot project of only eight sites serving 1029 drug users in 2004. Since then, it has rapidly expanded into a nationwide program covering 738 clinics and serving some 344,254 heroin users by the end of 2011, which accounted for approximately 30% of registered IDUs in China [7, 8]. The MMT program in China is believed to have made impressive progress in HIV infection and drug use among PWID [7] as a result of offering various ancillary services, including testing for HIV, syphilis and HCV, psychosocial support, and referrals for the treatment of HIV, tuberculosis and sexually transmitted diseases.

Because it borders the drug-trafficking route known as the ‘Golden Triangle’ and connects China with the Association of Southeast Asian Nations (ASEAN) countries, Guangxi Zhuang Autonomous Region (referred to hereafter as ‘Guangxi’) detected the first outbreak of HIV-1 infection among IDUs in 1996, and transmission through IDUs accounted for 69% of reported HIV cases in 2003, with the second-highest accumulated number of HIV cases in China since 2009 [9–11]. Guangxi therefore launched an MMT program in 2004 as one of the first eight national MMT pilot clinics [12], covering 72 clinics serving more than 30,000 heroin users by the end of 2014. Numerous studies conducted in China [13–16] showed that variations toward the prevalence of HIV and HCV infections differed dramatically across geographic locations. Nevertheless, the spatial distribution of these infections among MMT clients in border areas of China, such as Guangxi, is poorly understood, and most previously published studies [17–19] have concentrated on descriptive analysis. These findings could not visualize the geographic heterogeneity of these infections and detect the presence and location of a cluster in confined regions. We therefore undertook a spatial analysis of the prevalence of HIV and HCV infections among MMT clients from baseline data of treatment between 2004 and 2014, to characterize the geographic distribution patterns and co-clustered epidemic factors of HIV and HCV infections among drug injectors receiving MMT. This study might also have critical implications for policy-making and resource allocation based on the needs of each region, as well as for future MMT program implementation.

## Methods

### Study area

**Fig. 1:**
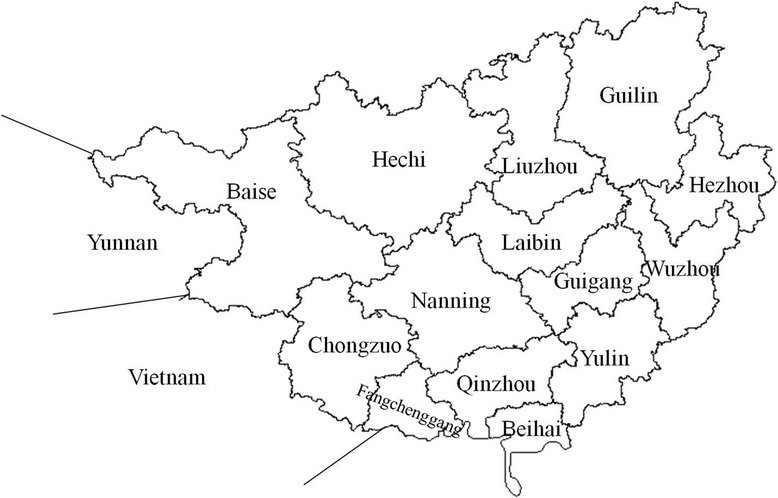
The location of Guangxi Autonomous Region, China

The study site is located in Guangxi, one of the areas most severely affected by HIV/AIDS in China, along with neighboring areas such as Vietnam and Yunnan (Fig. 1). Guangxi, which is located on the southwestern coast of China (104.26° ~ 112.04°E, 20.54° ~ 26.24°N), has an area of 236.7 thousand square kilometers and a population of approximately 52.82 million and encompasses 14 cities, 7 county level cities, 12 autonomic counties and 55 counties (Guangxi Statistical Yearbook in 2015). We examined the city-governed region and designated the others as county-level areas; there were thus 88 county-level areas in our study.

### Study population and data collection

Since the Chinese National Comprehensive AIDS Response Policy and the ‘Four Frees and One Care’ program (‘four frees’ refers to free HIV voluntary counseling and testing, free antiretroviral treatment for rural HIV patients and poor urban patients, free antiretroviral treatment for pregnant women living with HIV/AIDS, and free schooling for orphaned children of AIDS patients; ‘one care’ refers to financial subsidies for low-income AIDS patients and their families) were launched in 2003 [20], the Chinese Centers for Disease Control and Prevention (China CDC) has established national HIV databases. These cohort study databases can be used to study the prevalence of HIV and HCV infections among MMT clients, and details on the eligibility criteria to participate in the national MMT program have been reported elsewhere [7, 12, 21].

All clients attending the 72 MMT clinics of Guangxi from March 2004 to December 2014 were selected as our study population. Baseline data were collected upon inclusion in the MMT program using an interview questionnaire. Demographic characteristics focused on gender, residential address, ethnicity, marital status, occupation, education, and living status. Drug use questions focused on the initial age and length of drug use, current drugs injected and substances used, method of drug-taking, injection frequency, and syringe sharing. Biological data included urine morphine testing and HIV and HCV testing. Most of the data were self-reported by clients, such as demographic characteristics and drug use questions. Only MMT clients with both HIV or HCV infection and a residential address were analyzed.

A total of 35,387 records (35,008 clients) from 2004 to 2014 were obtained from the baseline database of the 72 MMT clinics in Guangxi. We excluded 3993 records without HIV or HCV testing results and another 379 repeated records; therefore, approximately 31,015 MMT clients were included in our study, which accounted for 88.59% (31,015/35,008) of all MMT clients. To exclude the possibility that the exclusion of clients unmasked correlations among the remaining observations, we compared included clients (*N* = 31,015) and excluded clients (*N* = 4372) for all variables, and no differences were found between these groups (not shown in this report).

### Laboratory methods

Urine samples were collected from all participants for morphine testing with colloidal gold diagnostics, and a positive urine result was indicative of a current heroin user. Blood samples were collected from all participants for HIV and HCV serologic testing with an enzyme-linked immune sorbent assay (ELISA). Western blot testing was conducted to confirm positive HIV ELISA results. All clients with HIV- and HCV-positive results received counseling and referral to the local China CDC, based on the city/county level of the client’s residence, for follow-up testing and treatment. Seronegative clients were also advised to proceed for follow-up testing in the future as clinically indicated.

### Statistical analysis

Spatial analysis was initiated by geolocating residential addresses of MMT clients using electronic maps of Guangxi (Bureau of Surveying and Mapping, Guangxi, China). We confirmed that all addresses were located within residential areas and not in non-residential locations, such as rail yards or industrial areas.

Baseline characteristics of the prevalence of HIV, HCV and co-infections were compared using chi-square tests, and the statistical analysis was performed using the SPSS Statistical Package for Social Sciences (SPSS Inc., Chicago, IL, USA). Geographic autocorrelation analysis was conducted using ArcGIS version 10.2 (ESRI Inc., Redlands, California, USA), and geographic scan statistics were performed using SaTScan™ v9.1.1 software (Martin Kulldorff together with information Management Services Inc., Boston, USA).

Geographic autocorrelation analysis was applied to describe the correlation of a single variable between pairs of neighboring observations, with the standard measure of the Moran statistic. First, global spatial autocorrelation was used to explore the distribution of infections, in which all counties were seen as a whole [22]. The values of Moran’s *I* ranged from +1 (indicating strong autocorrelation) to 0 (indicating a random pattern) to −1 (indicating over-dispersion and uniformity). Second, Local Indicators of Spatial Association (LISA) was applied to identify significant spatial outliers and generate four geographic patterns, including high-high, high-low, low-high and low-low [14]. A high-high pattern indicated that a county and its surroundings collectively had a higher infection rate than the average. A low-low pattern showed that a county and its surroundings collectively had a lower infection rate than the average. A high-low pattern indicated that a county with an above-average infection rate was surrounded by counties with below-average infection rates. A low-high pattern showed that a county with a below-average infection rate was surrounded by counties with above-average infection rates.

Geographic scan statistics were applied to test for the presence and location of clusters. This analysis imposes a circular window of varying radii on the map surface and allows its center to move, so that at any given position and size, the window includes different sets of adjacent neighboring areas. As the window is placed at each neighborhood center, its radius varies continuously, from zero to a maximum radius that never exceeds 50% of the total study population. The method allows the circular window to continuously vary in both location and size, thereby creating a large number of distinct circular clusters. The significance of the identified clusters was tested with a likelihood ratio test against a null distribution obtained from Monte Carlo simulations [23]. Details of how the likelihood function is maximized over all windows under the Poisson assumption have been described elsewhere [24–26]. For the Monte Carlo inference, 999 replications were performed for ordinal or nominal variables, and 9999 replications were performed for dichotomous variables. After a cluster was identified, the strength of the clustering was estimated using the relative risk of infections within the cluster versus outside the cluster. The null hypothesis of no clusters was rejected when the *P*-value was less than or equal to 0.05.

## Results

### Study population

Approximately 31,015 clients with valid data for both demographic characteristics and HIV or HCV testing results from 2004 to 2014 were obtained from the database of the 72 MMT clinics in Guangxi. The majority of clients were males (90.39% of the sample), unemployed (55.22%), Han ethnic groups (67.26%), had a junior secondary school education (62.75%) and were living with family or relatives (79.75%). In total, 48.45% reported never having been married, 43.12% were married, and 8.33% were divorced, separated or widowed. Most of the clients obtained their living expenses in the past 6 months from family or friends (53.46%), followed by casual wages (26.01%) and fixed wages (4.51%). The remaining portion of the sample obtained their living expenses from social welfare, criminal activity or other means. In terms of drug use, the average age of initial drug use and length of drug use were 24.18 ± 6.64 years and 8.91 ± 5.03 years, respectively. The main drug currently injected was heroin (87.70%), and 68.28% used drugs by injection only. The average frequency of drug use was 3.06 ± 1.25 times per day in the past month, and 23.25% self-reported that they had shared needles with others. Of all MMT clients at baseline, 56.05% (17,383/31,015) had positive urine morphine testing results, 13.05% (4046/31,015) were infected with HIV (including the number of individuals who were infected with HIV only and those who were HIV/HCV co-infected), 72.51% (22,488/31,015) were infected with HCV (including the number of individuals who were infected with HCV only and those who were HIV/HCV co-infected), and 11.96% (3708/31,015) were HIV/HCV co-infected. There were significant differences in terms of demographic and behavioral characteristics among HIV, HCV, and HIV/HCV co-infected clients (Table 1).

**Table 1:** Baseline characteristics of MMT clients with HIV, HCV and co-infections in Guangxi (2004 ~ 2014)

Characteristic	Total (*n* = 31,015)		HIV clients (*n* = 4046)		HCV clients (*n* = 22,488)		Co-infected clients (*n* = 3708)	
	No. of clients	Proportion (%)	No. of clients	Proportion (%)	No. of clients	Proportion (%)	No. of clients	Proportion (%)
Gender			χ^2^ = 37.75, *P* < 0.001		χ^2^ = 96.01, *P* < 0.001		χ^2^ = 118.25, *P* < 0.001	
Male	28,036	90.39	3550	87.74	20,101	89.39	3249	87.62
Female	2979	9.61	496	12.26	2387	10.61	459	12.38
Occupation			χ^2^ = 49.68, *P* < 0.001		χ^2^ = 505.12, *P* < 0.001		χ^2^ = 558.71, *P* < 0.001	
Unemployed	17,125	55.21	2415	59.69	13,143	58.44	2216	59.76
Farmers	9109	29.37	1135	28.05	5812	25.85	1034	27.89
Others	4781	15.42	496	12.26	3533	15.71	458	12.35
Ethnic groups			χ^2^ = 111.43, *P* < 0.001		χ^2^ = 419.45, *P* < 0.001		χ^2^ = 472.78, *P* < 0.001	
Han	20,860	67.26	3004	74.24	15,876	70.60	2779	74.95
Zhuang	9399	30.30	990	24.47	6093	27.09	880	23.73
Others	756	2.44	52	1.29	519	2.31	49	1.32
Marital status			χ^2^ = 144.04, *P* < 0.001		χ^2^ = 141.98, *P* < 0.001		χ^2^ = 242.64, *P* < 0.001	
Married	13,375	43.12	1426	35.25	9329	41.48	1305	35.19
Unmarried	15,055	48.54	2155	53.26	11,079	49.27	1972	53.18
Others	2585	8.34	465	11.49	2080	9.25	431	11.63
Education			χ^2^ = 74.10, *P* < 0.001		χ^2^ = 27.92, *P* < 0.001		χ^2^ = 89.74, *P* < 0.001	
Primary level education or below	8274	26.68	1276	31.54	6129	27.25	1168	31.50
Junior secondary school	19,461	62.75	2446	60.45	13,911	61.86	2238	60.36
Senior school or above	3280	10.57	324	8.01	2448	10.89	302	8.14
Living status			χ^2^ = 81.61, *P* < 0.001		χ^2^ = 183.78, *P* < 0.001		χ^2^ = 230.68, *P* < 0.001	
With family or relatives	24,734	79.75	3045	75.26	17,630	78.40	2796	75.40
With friends	971	3.13	108	2.67	644	2.86	96	2.59
Alone	2138	6.89	363	8.97	1623	7.22	321	8.66
Others	3172	10.23	530	13.10	2591	11.52	495	13.35
Living expense source in the past six months			χ^2^ = 103.70, *P* < 0.001		χ^2^ = 144.68, *P* < 0.001		χ^2^ = 212.56, *P* < 0.001	
From family or friends	16,582	53.46	2257	55.78	11,950	53.14	2051	55.31
From casual wages	8067	26.01	877	21.68	5623	25.00	805	21.71
From fixed wages	1399	4.51	117	2.89	986	4.39	108	2.91
Others	4967	16.02	795	19.65	3929	17.47	744	20.07
Age of initial drug use (years)			χ^2^ = 194.65, *P* < 0.001		χ^2^ = 223.97, *P* < 0.001		χ^2^ = 358.48, *P* < 0.001	
< 24	17,372	56.01	2677	66.16	13,180	58.61	2476	66.77
≥ 24	13,643	43.99	1369	33.84	9308	41.39	1232	33.23
Length of drug use at baseline (years)			χ^2^ = 1250.60, *P* < 0.001		χ^2^ = 3413.13, *P* < 0.001		χ^2^ = 4156.72, *P* < 0.001	
< 5	9763	31.48	411	10.16	5011	22.28	340	9.17
5–10	7655	24.68	922	22.79	5846	26.00	852	22.98
> 10	13,597	43.84	2713	67.05	11,631	51.72	2516	67.85
Route of drug use in the past six months			χ^2^ = 674.72, *P* < 0.001		χ^2^ = 6329.18, *P* < 0.001		χ^2^ = 6595.77, *P* < 0.001	
Injection intravenously only	21,177	68.28	3446	85.17	18,015	80.11	3209	86.54
Smoking or sniffing	7957	25.66	382	9.44	3055	13.58	297	8.01
Injection mixed with other	1881	6.06	218	5.39	1418	6.31	202	5.45
Receptive sharing of syringes with others			χ^2^ = 2840.14, *P* < 0.001		χ^2^ = 1566.26, *P* < 0.001		χ^2^ = 3753.07, *P* < 0.001	
Yes	7211	23.25	2276	56.25	6543	29.10	2137	57.63
No	23,804	76.75	1770	43.75	1,5945	70.90	1571	42.37
Frequency of drug use in the past month			χ^2^ = 208.72, *P* < 0.001		χ^2^ = 196.19, *P* < 0.001		χ^2^ = 195.95, *P* < 0.001	
0 times/day	2485	8.01	154	3.81	1615	7.18	143	3.86
1–3 times/day	18,386	59.28	2278	56.30	13,147	58.46	2077	56.01
4–6 times/day	8303	26.77	1289	31.86	6322	28.11	1220	32.90
> 6 times/day	1489	4.80	288	7.12	1188	5.29	245	6.61
Missing	352	1.14	37	0.91	216	0.96	23	0.62
HIV status at baseline			–		χ^2^ = 855.02, *P* < 0.001		–	
Positive	4046	13.05			3708	16.49		
Negative	26,969	86.95			18,780	83.51		
HCV status at baseline			χ^2^ = 855.02, *P* < 0.001		–		–	
Positive	22,488	72.51	3708	91.65				
Negative	8527	27.49	338	8.35				
HIV/HCV co-infection at baseline			–		–		–	
Double positive	3708	11.96						
Double negative	8189	26.40						
Single positive and negative	19,118	61.64						
Urine morphine testing results at baseline			χ^2^ = 24.79, *P* < 0.001		χ^2^ = 13.21, *P* = 0.001		χ^2^ = 33.50, *P* < 0.001	
Positive	17,383	56.05	2442	60.36	12,622	56.13	2190	59.06
Negative	13,085	42.19	1562	38.61	9507	42.28	1445	38.97
Missing	547	1.76	42	1.04	359	1.59	73	1.97

**Fig. 2:**
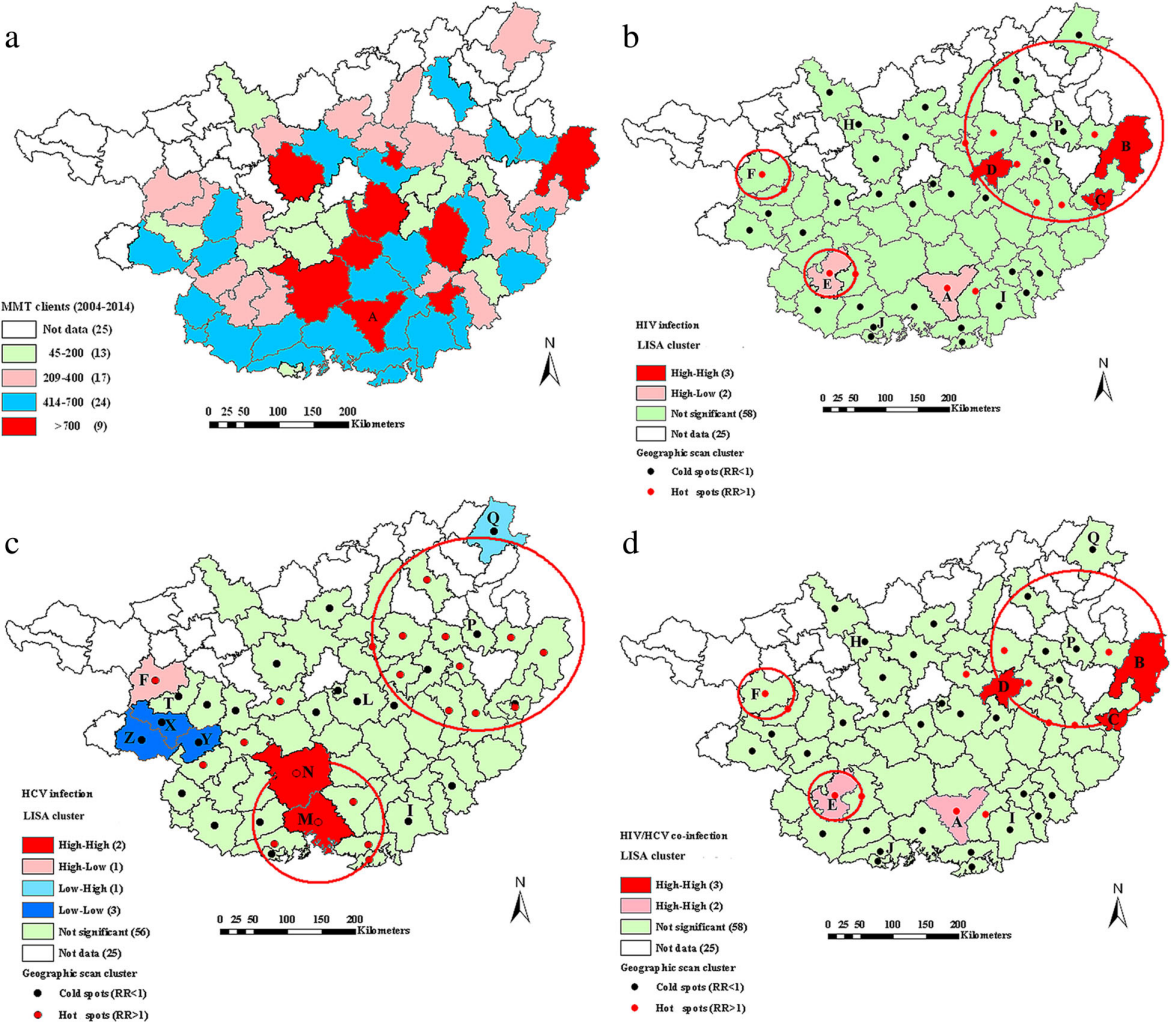
Distribution, LISA cluster map and geographic scan clusters of HIV, HCV and co-infections of MMT clients from 2004 to 2014 in Guangxi. Red circles represent high risk clusters. **a** Distribution of all MMT clients; **b** Spatial clusters of HIV infection; **c** Spatial clusters of HCV infection; **d** Spatial clusters of HIV/HCV co-infection

Of the 88 county-level areas in Guangxi, 63 locations were covered by MMT clinics, with the cumulative number of clients ranging from 45 to 2634. Clinics with more than a cumulative number of 400 clients were mostly distributed in the southern areas, with A county as the center. A county accounted for 41.55% of the total clients in these clinics (Fig. 2a). The residential addresses for all MMT clients were distributed throughout Guangxi in a pattern that was more closely clustered than random (Moran’s *I* = 0.14, *P* = 0.022).

### Spatial clusters of HIV infection

The HIV infection rate of MMT clients was found to be strongly geographically clustered (Moran’s *I* = 0.33, *P* < 0.001). For high HIV infection rates, the results of LISA were similar to those of the geographic scan statistic (Fig. 2b and Table 2). LISA analysis identified three high-high locations distributed in B and C cities and D county and two high-low locations in A and E counties, which included 2844 MMT clients and 1041 HIV cases, with an HIV infection rate of 36.60% (1041/2844). Apart from these clusters, there were only 3005 HIV cases, with a 10.67% (3005/28,171) infection rate. The geographic scan statistic identified four significant high-risk clusters, which included 19 locations with a 23.73% (2264/9542) infection rate. The high-risk clusters were mainly concentrated in the northeastern parts where P county was the cluster center, along with the surrounding areas of E and F cities or A county alone. Apart from these clusters, there were only 1782 HIV cases with an 8.30% (1782/21,473) infection rate. For low HIV infection rates, the geographic scan statistic identified three significant low-risk clusters, which included 19 locations with a 3.66% (334/9131) infection rate. These low-risk areas were mainly distributed in the northwestern parts, where H city was the cluster center, or in the southern parts, where I county and J city were the cluster centers. LISA analysis showed that there were no significant low-low locations for HIV infection rates.

**Table 2:** General description of the clusters with high and low prevalence of HIV, HCV and co-infections among MMT clients in Guangxi (2004 ~ 2014)

Type of infection	Type of cluster	No. of counties/cities	Radius (km^2^)	MMT clients	No. of infections	Relative risk	Log likelihood ratio	*P* value
HIV infection	High risk cluster	14	11.95	7074	1405	1.80	147.72	<0.001
		2	6.24	627	220	2.79	81.90	<0.001
		2	6.14	708	173	1.91	28.76	<0.001
		1	0.00	1133	466	3.43	230.27	<0.001
	Low risk cluster	7	11.23	2556	74	0.21	156.90	<0.001
		7	8.91	4209	144	0.24	234.95	<0.001
		5	9.29	2366	116	0.36	84.01	<0.001
HCV infection	High risk cluster	14	11.95	7074	5626	1.13	30.50	<0.001
		8	9.48	6692	5655	1.22	81.59	<0.001
	Low risk cluster	12	12.01	4224	2392	0.75	90.92	<0.001
		4	7.61	1142	684	0.82	13.80	0.002
		1	0.00	680	381	0.77	14.11	0.008
		1	0.00	342	5	0.02	224.78	<0.001
HIV/HCV co-infection	High risk cluster	13	10.95	4888	1008	2.00	156.03	<0.001
		2	6.24	627	201	2.78	74.43	<0.001
		2	6.14	708	160	1.93	27.31	<0.001
		1	0.00	1133	466	3.43	230.27	<0.001
	Low risk cluster	7	8.91	4209	129	0.23	219.66	<0.001
		7	11.32	2556	68	0.21	143.51	<0.001
		5	9.29	2366	102	0.34	81.52	<0.001
		1	0.00	342	0	0.00	25.11	<0.001

### Spatial clusters of HCV infection

Significant spatial clustering was detected for the HCV infection of MMT clients (Moran’s *I* = 0.41, *P* < 0.001). For high HCV infection rates, LISA analysis observed two high-high locations distributed in M and N cities and one high-low location in F city, which included 3490 MMT clients and 3105 HCV cases, with an HCV infection rate of 88.97% (3105/3490). Apart from these clusters, there were only 19,383 HCV cases, with a 70.72% (19,383/27,525) infection rate. The geographic scan statistic identified two significant high-risk clusters, which included 22 locations with an 81.95% (11,281/13,766) infection rate. One cluster included eight locations distributed in southern areas where M city was the cluster center, and the other cluster included 14 locations distributed in the northeastern parts where P county was the cluster center. Apart from these clusters, there were only 11,207 HCV cases, with a 64.97% (11,207/17,249) infection rate. For low HCV infection rates, LISA analysis observed three low-low locations distributed in X, Y, and Z counties and one low-high location in Q county, with a 29.42% (373/1268) infection rate. The geographic scan statistic identified four significant low-risk clusters, which included 18 locations with a 54.20% (3462/6388) infection rate. These low-risk areas were mainly located in western parts, where T county was the cluster center, and in the central parts, where L city was the cluster center, along with sporadic counties, such as I and Q counties (Fig. 2c and Table 2).

### Spatial clusters of HIV/HCV co-infection

The co-infection rate of HIV/HCV among MMT clients was more closely clustered than resembling a random pattern (Moran’s *I* = 0.30, *P* = 0.003). The clustering pattern was closely similar to that of the HIV infection rate (Fig. 2d and Table 2). Except for Q county, the clustering areas for co-infection rates detected by the geographic scan statistic were identical with those of LISA analysis. The geographic scan statistic identified four significant high-risk clusters, with a 24.95% (1835/7356) co-infection rate of 18 locations, and four significant low-risk clusters, with a 3.16% (299/9473) co-infection rate of 20 locations.

### Co-clustering of HIV, HCV and co-infections

**Fig. 3:**
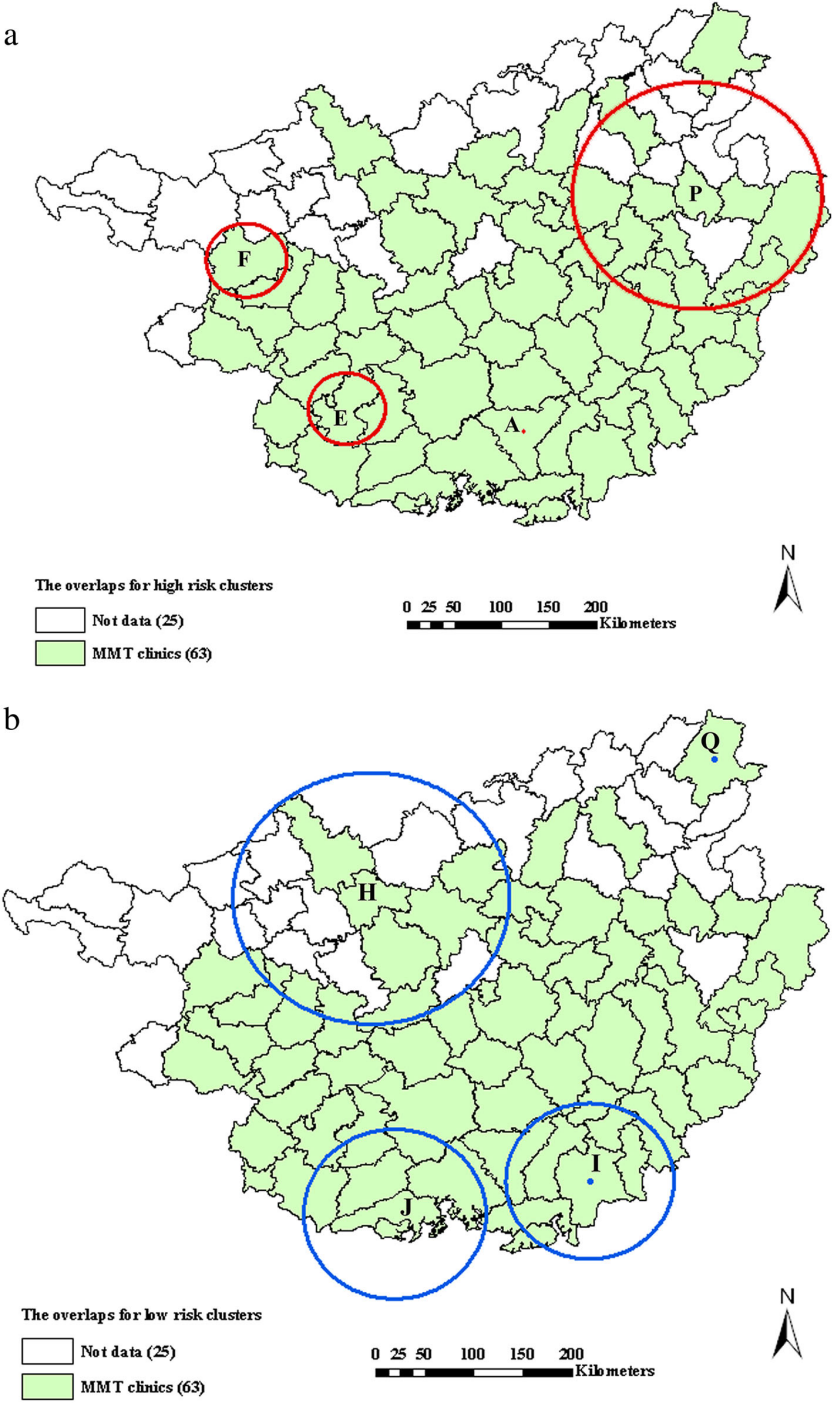
Co-clustering of HIV, HCV and co-infections among MMT clients from 2004 to 2014 in Guangxi. Red circles represent high risk clusters, blue circles represent low risk clusters. **a** High risk clusters; **b** Low risk clusters

As shown in Fig. 3, many significant clusters of HIV, HCV, or co-infections overlapped. For the significant high-risk clusters, the overlaps for these infections were located in the northeastern parts where P county was the cluster center, which covered 13 locations where the radius was 10.95 km^2^. In addition, the overlaps for HIV and co-infections were also located in the surrounding areas where E and F cities were the cluster centers, as well as A county (Fig. 3a). For the significant low-risk clusters, the overlap for HIV, HCV and co-infections was only I county, while the overlaps for HIV and co-infections were also located in the surrounding areas where H and J cities were the cluster centers, and the overlap for HCV and co-infections also contained Q county (Fig. 3b).

### Spatial clusters of epidemic factors

**Fig. 4:**
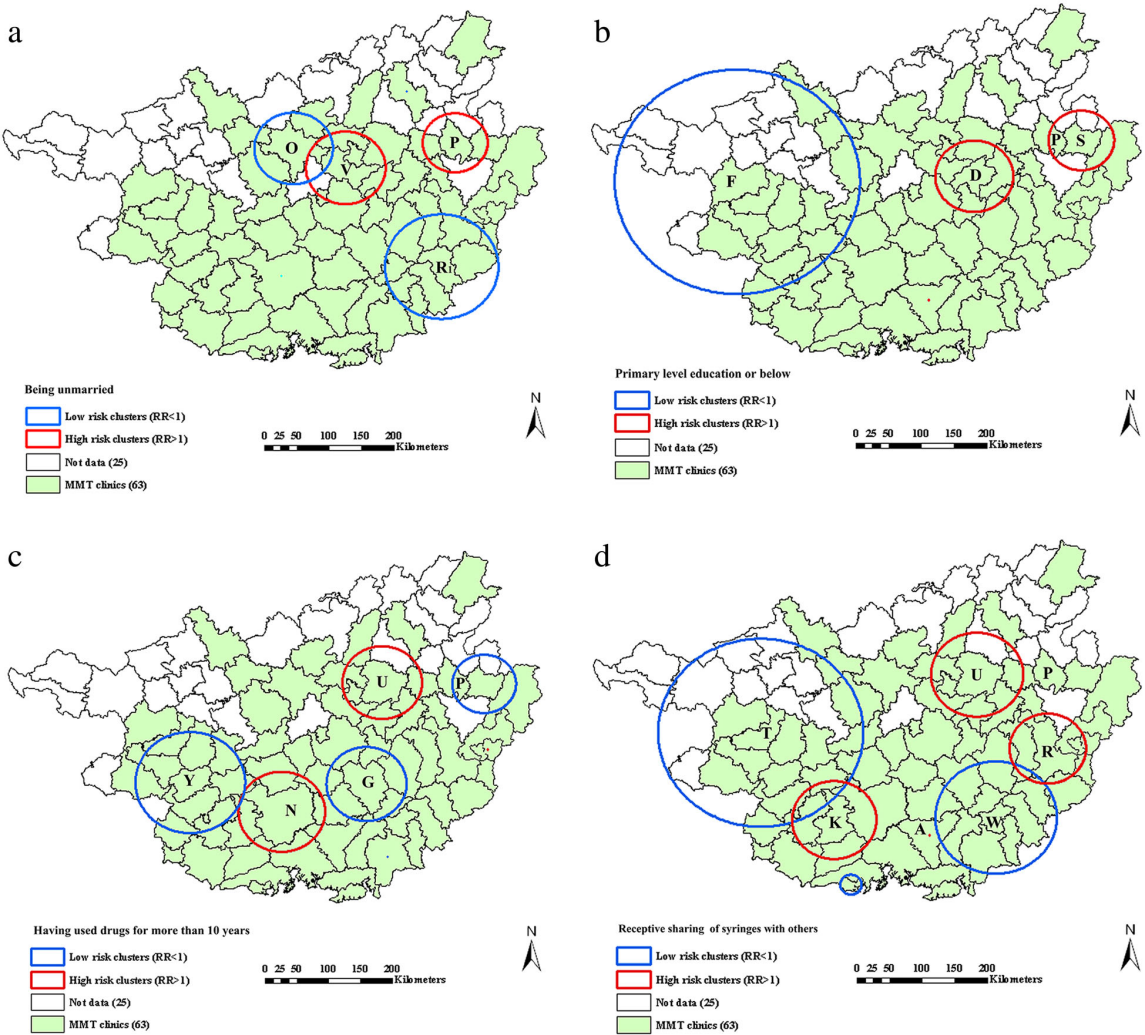
Distribution and significant spatial clustering of demographic and behavioral variables. **a** Being unmarried; **b** Primary level of education or below; **c** Having used drugs more than 10 years; **d** Receptive syringe sharing with others

Significant spatial clustering was also detected for several of demographic and behavioral factors, and most of them were likely to reside within the clusters of HIV, HCV, or co-infections (Fig. 4 and Table 3). Of the demographic factors, only being unmarried and having a primary level education or below were geographically clustered. Of risky injection practices, injectors who reported more than 10 years of drug use and receptive sharing of syringes with others were geographically clustered. As shown in Fig. 4, the high-risk clusters for these significant clustering characteristics were mainly located within or surrounding the northeastern parts where P county was the cluster center. In addition, two characteristics of injection practices were also located near one of the overlaps for HIV and co-infections (such as the surrounding areas where E city was the cluster center) or one of the high-risk clusters of HCV infection (such as the southern parts where M city was the cluster center). For the low-risk clusters, most of them were distributed in the western and southeastern parts, which are located near one of the low-risk clusters for HIV, HCV, or co-infections.

**Table 3:** Significant high risk clusters of demographic and behavioral characteristics of MMT clients in Guangxi

Characteristic	Moran’s *I* ± SD expected *I* = −0.0161	*P* value for clustering	No. of counties/cities	Radius (km^2^)	Relative risk
Being unmarried	0.2002 ± 0.0024	<0.001	5	7.50	1.38
			3	6.77	1.21
Primary level education or below	0.1167 ± 0.0024	0.007	4	7.40	1.43
			2	6.77	1.48
Having used drugs for more than 10 years	0.0979 ± 0.0024	0.020	3	7.93	1.63
			3	7.56	1.69
Receptive sharing of syringes with others	0.1091 ± 0.0024	0.010	6	8.03	1.62
			4	7.73	1.34
			3	7.34	1.74
			1	0.00	1.99

## Discussion

To our knowledge, this is the first study to investigate the spatial distribution patterns of HIV and HCV epidemics in relation to MMT clients and their possible interactions in Guangxi. The overall infection rates of HIV, HCV, and HIV/HCV among MMT clients at treatment baseline were 13.05%, 72.51% and 11.96% respectively, which were similar to the rates of high-transmission areas (including Yunnan, Guizhou, Sichuan and Xinjiang) of China, but distinctly higher than those of the other provinces [8]. This finding indicated that the rates of these infections remained highly concentrated among provinces along the traditional drug-trafficking routes, and MMT clinics have recruited more HIV- or HCV-infected drug users as a result of the 2006 national policy to relax the eligibility criteria for MMT enrollment. The similar infection rates of HIV and HIV/HCV suggest that HIV-infected MMT clients were at high risk of co-infected HCV infection. Additionally, this finding showed the important role of IDUs in driving the HCV epidemic among PWID and HIV-infected individuals, which was consistent with previously published evidence [3, 6, 27].

Our study demonstrated that HIV, HCV and co-infections in Guangxi all exhibited significant geographic clustering at the county level, and their distribution patterns overlapped to some degree, particularly for HIV and HIV/HCV co-infection. The most significant high-risk overlapping clusters for these infections surrounded P county in the northeastern area of Guangxi. The overlaps for HIV and co-infections were also located in the area surrounding E and F cities in the southwestern areas of Guangxi, as well as A county. Several important points are considered in interpreting the geographic distribution patterns of these infections. First, E city is adjacent to Vietnam, where the HIV epidemic was driven by IDUs in the early phase and formed the spreading trends from the South to the North since 1990. Guangxi thus first detected a domestic HIV-1 case among IDUs in one county of E city in 1996, which gradually led to an HIV outbreak among drug users in the surrounding border areas [9, 28, 29]. Second, because of its shared border with the ‘Golden Triangle’ and Yunnan province, which were the earliest and most severe HIV/AIDS epidemic areas in China, F city became one of the most severely affected HIV/AIDS areas initially fueled by IDUs in Guangxi [30]. Third, as previous studies have shown, the geographic concentrations of HIV in poor and underserved areas and the dispersion of HIV along roads and highways [31–34] are high, and it is indeed the case that most of the locations surrounding P county, including E and F cities, belong to economically depressed areas. These findings corroborated that HIV and HCV epidemics first broke out within the border areas adjacent to the ‘Golden Triangle’ and then along drug trafficking routes to other parts of Guangxi, and poverty might play an important role in accelerating these epidemics. Meanwhile, the most significant low-risk overlapping clusters for HIV, HCV or co-infections were mainly located in areas surrounding H and J cities or I county, which was consistent with the trend from routine monitoring data of Guangxi. This information suggested that some behavioral or biological protective factors appear to have slowed the transmission of these infections in the low-risk clusters.

The distribution patterns of HIV, HCV and co-infections are similar to those across different counties, which might be attributed to the similarities in the features of the epidemics. Compared with related studies [35–37], our findings showed that several epidemic factors, such as being unmarried, having a primary level of education or below, having used drugs for more than 10 years and receptive sharing of syringes with others, were geographically clustered. Most of the individuals with these factors were more likely to reside in one of clusters for HIV, HCV and co-infections, especially for the high-risk clusters (e.g., the areas surrounding P county as the cluster center). Numerous studies have reported that the emergence of MMT and other harm reduction programs have resulted in lower levels of risky behaviors and reductions in the HIV epidemic [7, 12, 38, 39]. However, not all previous studies have found such a tight linkage, as one study from the San Francisco Bay Area [40] found that risky injection practices were indeed lower among drug users from poorer communities targeting harm reduction programs, but the prevalence of HIV remained high. Therefore, additional studies should be conducted to evaluate the spatial distribution of these infections and their association with epidemic features after MMT programs have been more widely established.

The similar prevalence and overlapping spatial clusters found in our study between HIV and HIV/HCV co-infection suggest a higher prevalence of HCV co-infection among HIV-infected PWID in Guangxi. Co-infection of HIV and HCV interact synergistically by affecting the transmission history and reducing the immune clearance of the other. Individuals co-infected with HCV could boost the occurrence of HIV infection, with the perinatal transmission risk doubling in HIV-infected mothers [41, 42], thus altering immunological responses to antiretroviral therapy and accelerating the risk of drug-related hepatotoxicity and consequently cirrhosis, liver failure, and hepatocellular carcinoma [43–45]. Meanwhile, HIV-infected individuals without treatment are less likely to spontaneously clear HCV infection; they may then experience more rapid HCV disease progression than HIV-negative individuals [3, 46]. Given the fact that a large proportion of HIV cases are acquired through IDUs in Guangxi, there is an urgent need to comply with international guidelines that recommend HCV screening for HIV-infected individuals, investment in building HCV surveillance and care, and access to direct-acting antiviral treatment for those with chronic active infection [47–50]. Comprehensive measures in parallel to MMT, such as antiretroviral therapy, 100% use of condoms, needle exchanges and sexually transmitted disease management services should be promoted as well.

Several limitations in this study should be noted. First, it is difficult to determine whether the distribution patterns and associations of the MMT clients described in this report are true for the entire population of injectors in this region. However, one study reported by national sentinel surveillance has shown that the national HIV prevalence among MMT clients was not significantly different from that of non-MMT drug users from 2004 to 2009 [8]. Second, most of the data included were obtained from 2008 to 2014, given that a national MMT program database was developed in 2004 to monitor the pilot and was later upgraded to a web-based management database in 2008. Third, most of the data, including residential addresses and drug use behaviors, were self-reported, and we have no way knowing the proportion of the clients giving false or misleading information. Nevertheless, the information bias might be narrowed given that the staff members of local MMT clinics needed to receive a series of professional trainings on assessment surveys, after which they could upload clients’ information to the national web-based management database prior to their assignment. Most of the clients were later contacted at the addresses they provided as well. Finally, MMT clients without a fixed address (and who were thus more likely to be at high risk) were excluded, and drug use is likely to occur in venues or areas of the city that might not coincide with an address. Therefore, the location of consumption would have been a better approach for the analysis.

## Conclusions

Using spatial analysis for detecting the HIV and HCV epidemics among drug users from a cohort study of MMT programs from 2004 to 2014, we revealed two important findings. First, HIV, HCV and co-infections among MMT clients in Guangxi Zhuang Autonomous Region all presented substantial geographic heterogeneity at the county level with a number of overlapping significant clusters. Second, areas surrounding P county were effective in enrolling high-risk clients in their MMT programs, which in turn might allow PWID to inject less, share fewer syringes, and receive referrals for HIV or HCV treatment in a timely manner.

## Abbreviations

AIDS: Acquired Immune Deficiency Syndrome
ASEAN: The Association of Southeast Asian Nations
CDC: Center for Disease Control and Prevention
ELISA: Enzyme-li﻿nked immune sorbent assay
GIS: Geographical information system
HCV: Hepatitis C virus
HIV: Human immunodeficiency virus
IDUs: Injecting drug users
LISA: Indicators of Spatial Association
MMT: Methadone maintenance treatment
PWID: People who inject drugs
SPSS: The SPSS Statistical Package for Social Sciences
